# Effects of cadmium stress on physiological indexes and fruiting body nutritions of *Agaricus brasiliensis*

**DOI:** 10.1038/s41598-021-87349-x

**Published:** 2021-04-21

**Authors:** Lingyun Liu, Hua Chen, Jing Yuan, Yixiang Wang, Boqi Weng, Penghu Liu, Guoxue Li

**Affiliations:** 1 College of Horticulture, Fujian Agriculture and Forestry University, Fuzhou, 350002 Fujian China; 2 National Engineering Research Center of JUNCAO Technology, Fujian Agriculture and Forestry University, Fuzhou, 350002 Fujian China; 3 Institute of Agricultural Ecology, Fujian Academy of Agriculture Sciences, Fuzhou, 350003 Fujian China; 4 Fujian Key Laboratory of Agro-Ecological Processes in Red Soil Hilly Region, Fuzhou, 350003 Fujian China; 5 Beijing Key Laboratory of Farmland Soil Pollution Prevention and Remediation, College of Resources and Environmental Science, China Agricultural University, Beijing, 100193 China

**Keywords:** Biochemical assays, Nutrition

## Abstract

In this study, 0, 0.5, 1, 1.5, 2, 4, 6 and 8 mg·kg^−1^ of cadmium were added to the cultivation materials. In order to study the effects of different concentrations of Cd stress on J1 and J77, the contents of antioxidant enzymes, proline and malondialdehyde, Cd content, agronomic traits and yield of fruiting bodies of *Agaricus brasiliensis* were determined, and the nutritional components such as polysaccharide, triterpene, protein, total sugar and total amino acid were determined. The results showed that the physiological indexes of strain J1 and J77 changed regularly under different concentrations of Cd stress. J1 was a high absorption and low tolerance variety, while J77 was a low absorption and high tolerance variety. Low concentration of Cd promoted the growth of strain J1, and higher concentration of Cd promoted the growth of strain J77. The contents of protein and total amino acids in the two strains changed greatly, followed by polysaccharides, which indicated that Cd stress had the greatest impact on the three nutrients, and other nutrients were not sensitive to Cd stress.

## Introduction

*Agaricus brasiliensis* is an edible mushroom which has attracted much attention because of its good therapeutic effect on cancer, hypertension, arteriosclerosis, heart disease, diabetes and chronic hepatitis^1^. Heavy metal pollution is one of the most urgent issues to be solved in food security and human health^2^. Cd was one of the most common pollutant among all the heavy metals identified in soil and crops^3^. Fungi can absorb metals and metalloids because mycelium can capture and bioaccumulate these heavy metals and other trace elements in the growth matrix^4^. Therefore, the Cd content in some edible fungus fruiting bodies exceeded the edible standard^5,6^. *A. brasiliensis* mycelium has strong ability to accumulate Cd, which has attracted many scholars' attention^7–9^.

Cd can not only inhibit or destroy many kinds of cells and physiology, but also cause oxidative stress and antioxidant reaction^10^. The plants fed with Cd exhibited a decline in growth, the levels of carbonic anhydrase and chlorophyll pigments and net photosynthetic rate^11^. Heavy metal exposure has been observed to have a plethora of detrimental effects on plant physiology such as stunted growth, chlorosis, reduced biochemical and metabolomic functions, low yields and poor fruit quality^12^. Under Cd stress, plants have a series of changes, and medicinal plants increase the production and accumulation of terpenoids, alkaloids and phenols^13^. Cd stress reduced growth, photosynthetic pigment, ascorbic acid, catalase (CAT) activity and yield^14^.

Accumulation of Cd in plants has been studied thoroughly^15^, but there are a few studies on Cd accumulation in edible fungi^16^. It was reported that the polysaccharide had strong adsorption capacity to heavy metal ions (Cd, Cu, Zn and Pb), the accumulation of Cd in the fruiting body of *Boletus edulis* was high^17^. The response of *A. brasiliensis* under Cd stress mainly focused on physiology, changing cultivation measures and transcriptomics research^18^. The results showed that the yield of *A. brasiliensis* decreased under high concentration Cd stress, and with the increase of Cd concentration, the Cd content in fruiting bodies also showed an upward trend^19^. It was observed that the antioxidant enzyme activities and fruiting body nutritions of *A. brasiliensis* were changed with the stress^20^. *A. brasiliensis* has edible and medicinal value, so it is necessary to study the effect of Cd stress on its nutritions.

Through 20 years of research on the Cd-enrichment regulations of *A. brasiliensis*, we found that different strains had different capacity of Cd accumulation and J1 was a variety with low Cd tolerance and high Cd accumulation, while J77 was a variety with high Cd tolerance and low Cd accumulation^21^. In the early stage of our group of research, the Cd-rich agronomic traits of *A. brasiliensis* and the transcriptomics of hyphae were studied. Studies have shown that there are differences among the genotypes of different strains, which may be due to the molecular mechanism regulating the genotypes^22^. The main research contents of this study are: (1) The influence of Cd on agronomic traits including yield, stipe length, etc., (2) The influence of Cd on the antioxidant system, (3) The influence of Cd on the nutritions of fruiting bodies. The purpose of the research is to systematically reveal the effect of Cd on the antioxidant system of *A. brasiliensis* of different genotypes (different Cd absorption and tolerance) and the effect on nutritions, laying the foundation for food safety and nutritions evaluation.

## Results

### Effects of Cd stress on agronomic characters and yield of fruiting bodies

The mature but closed hats fruiting bodies of *A. brasiliensis* were collected, and the mycelium and cultivated soil at the bottom were treated, and the agronomic traits and yield were measured. It can be seen from Table 1 that Cd concentration has a great impact on the weight of single mushroom of J1 fruiting body. With the increase of Cd concentration, the weight of single mushroom decreased, and the difference between treatments were significant. Among them, the weight of single mushroom under the control of J1 was the heaviest, which was 41.55% higher than that of 8 mg kg^−1^ Cd treatment. The number of J1 fruiting bodies was the most in 4 mg kg^−1^ treatment, which was 5.71 times higher than that in 8 mg kg^−1^ treatment. Combined with the weight and the number of fruiting bodies, they showed a certain proportion under non severe stress. Although the weight of single mushroom in the control treatment of J1 was heavier than that of other treatments, and its number was much lower than that of the treatment of 4 mg kg^−1^. This indicates that the weight of single mushroom is proportional to the number of fruiting bodies to a certain extent, and the formation of this relationship may be affected by the nutrients of the cultivated materials. From the length of strains, it can be seen that the differences among the treatments are not significant. Under the treatment of 8 mg kg^−1^, the height of J1 was higher. There was no difference in stipe length among different treatments, which indicated that the stipe length and pileus thickness of J1 were relatively stable and were not affected by Cd concentration stress. The diameter of pileus and stipes directly determines the weight of pileus and stipes. Cd stress had a great effect on the weight and number of single mushroom of J1, which decreased first, then increased with the concentration of Cd.

**Table 1:** Effects of different concentrations of cadmium stress on the agronomic characters of J1.

Treatment	J1 (CK0)	J1 (0.5)	J1 (1.0)	J1 (1.5)	J1 (2.0)	J1 (4.0)	J1 (6.0)	J1 (8.0)
Average weight (g)	15.67 ± 0.17a	15.09 ± 0.23b	13.98 ± 0.04c	12.98 ± 0.10d	12.57 ± 0.27de	13.77 ± 0.38c	12.40 ± 0.08e	11.07 ± 0.21f
Number	16 ± 0.94de	21 ± 1.25c	35 ± 1.70b	15 ± 0.82e	22 ± 0.94c	40 ± 1.70a	21 ± 0.47c	7 ± 0.47f
Strain height (cm)	7.94 ± 0.04a	7.83 ± 0.21ab	7.09 ± 0.39bc	7.13 ± 0.38bc	7.78 ± 0.22ab	7.74 ± 0.34ab	6.69 ± 0.20c	7.57 ± 0.52ab
Cap diameter (cm)	3.56 ± 0.11ab	3.83 ± 0.17a	3.35 ± 0.11bc	3.55 ± 0.18ab	3.44 ± 0.10b	3.31 ± 0.08bc	3.46 ± 0.28b	3.03 ± 0.05c
Stipe diameter (cm)	1.47 ± 0.07a	1.40 ± 0.08ab	1.42 ± 0.10ab	1.23 ± 0.06c	1.31 ± 0.07ab	1.33 ± 0.08ab	1.36 ± 0.18ab	1.20 ± 0.08c
Stipe length (cm)	6.62 ± 0.07a	6.27 ± 0.45a	5.88 ± 0.51a	6.00 ± 0.41a	6.46 ± 0.52a	6.75 ± 0.29a	5.91 ± 0.23a	6.57 ± 0.33a
Cap thickness (cm)	2.51 ± 0.04a	3.07 ± 0.45a	2.47 ± 0.28a	3.28 ± 1.08a	2.49 ± 0.07a	2.55 ± 0.10a	2.48 ± 0.19a	2.30 ± 0.22a
Cap weight (g)	8.70 ± 0.20a	7.72 ± 0.25ab	7.32 ± 0.53b	8.13 ± 0.24ab	7.37 ± 0.19b	7.88 ± 0.38ab	7.50 ± 0.88b	6.13 ± 0.34c
Stipe weight (g)	7.06 ± 0.04a	7.36 ± 0.45a	6.68 ± 0.49ab	4.85 ± 0.32c	5.18 ± 0.18c	5.89 ± 0.05bc	4.90 ± 0.96c	4.93 ± 0.42c

Different lowercase letters in the same column indicate significant difference (p < 0.05).

It can be seen from Table 2 that the weight of single mushroom in these 8 treatments is significant, but the regularity is not very obvious. The number of mushrooms increased with the increase of Cd concentration. The number of mushrooms in 8 mg kg^−1^ treatment of J77 was the biggest, which was more than twice that of other treatments. This indicated that high concentration of Cd stress promoted the growth of J77 fruiting bodies. The size of J77 did not change with the increase of Cd concentration. From the cap diameter, we can see that there is no difference among the first seven treatments, but there is a significant difference between 8 mg kg^−1^ treatment of J77 and other treatments. However, the stipe diameter of J77 control treatment was significantly lower than that of the latter 7 treatments, and the difference was significant. There was no significant difference in stipe length among different treatments, which was regulated by J77 variety specificity, while Cd stress did not affect stipe length. There was a significant difference in the cap thickness of J77 between 2 mg kg^−1^ Cd treatment and other treatments, but there was no significant difference among other treatments. The weight of cap and stipe affected the weight of single mushroom. The weight of cap and stipe was light, and the weight of single mushroom was also low.

**Table 2:** Effects of different concentrations of cadmium stress on the agronomic characters of J77.

Treatment	J77 (CK)	J77 (0.5)	J77 (1.0)	J77 (1.5)	J77 (2.0)	J77 (4.0)	J77 (6.0)	J77 (8.0)
Average weight (g)	13.91 ± 0.85b	17.39 ± 0.85a	17.95 ± 0.52a	14.54 ± 1.43b	14.48 ± 0.35b	14.27 ± 1.51b	17.00 ± 1.64a	13.80 ± 0.51b
Number	32 ± 2.05c	28 ± 2.94cd	38 ± 3.09b	24 ± 1.41de	28 ± 3.27cd	20 ± 1.63e	33 ± 2.16bc	72 ± 2.62a
Strain height (cm)	6.13 ± 0.43a	5.19 ± 0.70a	5.28 ± 0.35a	5.41 ± 0.39a	5.69 ± 0.44a	5.57 ± 0.56a	5.44 ± 0.41a	5.39 ± 0.42a
Cap diameter (cm)	3.80 ± 0.16abc	4.06 ± 0.19a	4.00 ± 0.01ab	3.67 ± 0.10bc	3.71 ± 0.06abc	3.79 ± 0.14abc	3.82 ± 0.16abc	3.63 ± 0.22c
Stipe diameter (cm)	1.35 ± 0.04e	2.37 ± 0.20a	2.38 ± 0.24a	1.86 ± 0.07bcd	1.61 ± 0.17de	1.70 ± 0.13cd	2.07 ± 0.04ab	2.03 ± 0.22abc
Stipe length (cm)	5.34 ± 0.36a	4.44 ± 0.49a	4.61 ± 0.36a	4.54 ± 0.35a	4.98 ± 0.44a	4.71 ± 0.47a	4.68 ± 0.32a	4.61 ± 0.44a
Cap thickness (cm)	2.02 ± 0.11a	1.86 ± 0.15ab	1.79 ± 0.02b	1.87 ± 0.02ab	1.91 ± 0.07ab	1.85 ± 0.13ab	1.87 ± 0.11ab	1.80 ± 0.04ab
Cap weight (g)	8.99 ± 0.72bc	10.66 ± 1.24ab	10.92 ± 0.33a	8.87 ± 0.98bc	9.18 ± 0.21abc	9.29 ± 0.86abc	10.48 ± 0.98ab	8.10 ± 0.66c
Stipe weight (g)	4.88 ± 0.21c	6.70 ± 0.38a	7.02 ± 0.20a	5.67 ± 0.57bc	5.25 ± 0.21c	4.98 ± 0.65c	6.52 ± 0.66ab	5.70 ± 0.20bc

Different lowercase letters in the same column indicate significant difference (p < 0.05).

**Figure 1:**
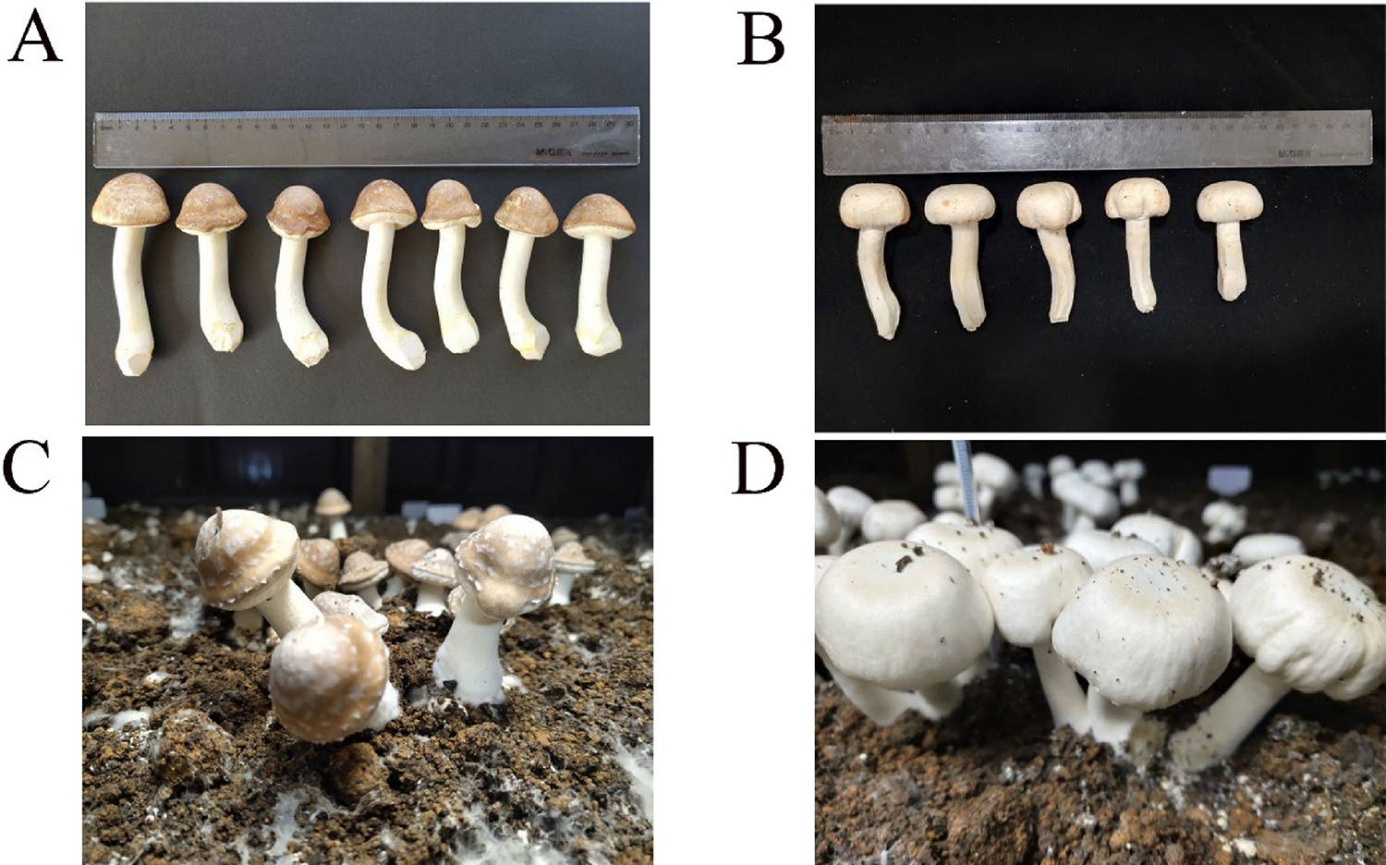
(**A**) The shape of J1’s fruiting body; (**B**) The shape of J77’s fruiting body; (**C**) The form of J1’s fruiting body during cultivation; (**D**) The form of J77’s fruiting body during cultivation.

The results showed that the strains of both cultivars were highly specific, and J1 was the cultivar with bigger fruiting bodies, while J77 was the cultivar with smaller fruiting bodies. The weight of J1 is lighter than J77. It can be seen from Fig. 1A that J1 is a brown umbrella shaped cap, while the shape of J77 in Fig. 1B is similar to *A. bisporus*, and the cap is white cap. It can be seen from Fig. 1C that the growth of J1 is slightly sparse, while the growth of J77 in Fig. 1D is denser and the number of fruiting bodies is higher. The number of fruiting bodies of J77 was significantly higher than J1, which indicated that J77 grew better than J1. Under the same concentration of Cd stress, J77’s tolerance was significantly higher than J1, indicating that J77 had stronger Cd tolerance than J1. The pileus diameter of J77 was generally bigger than J1, and the stipe diameter also showed such regularity. The thickness of J1 was larger than J77, but the weight of J1 was lower than J77. The stipe of J1 was longer and solid, and J77 was shorter and hollow. Based on the above agronomic traits and figures, it can be seen that the statistical agronomic traits are corresponding to the growth status and fruiting body morphology of J1 and J77.

**Figure 2:**
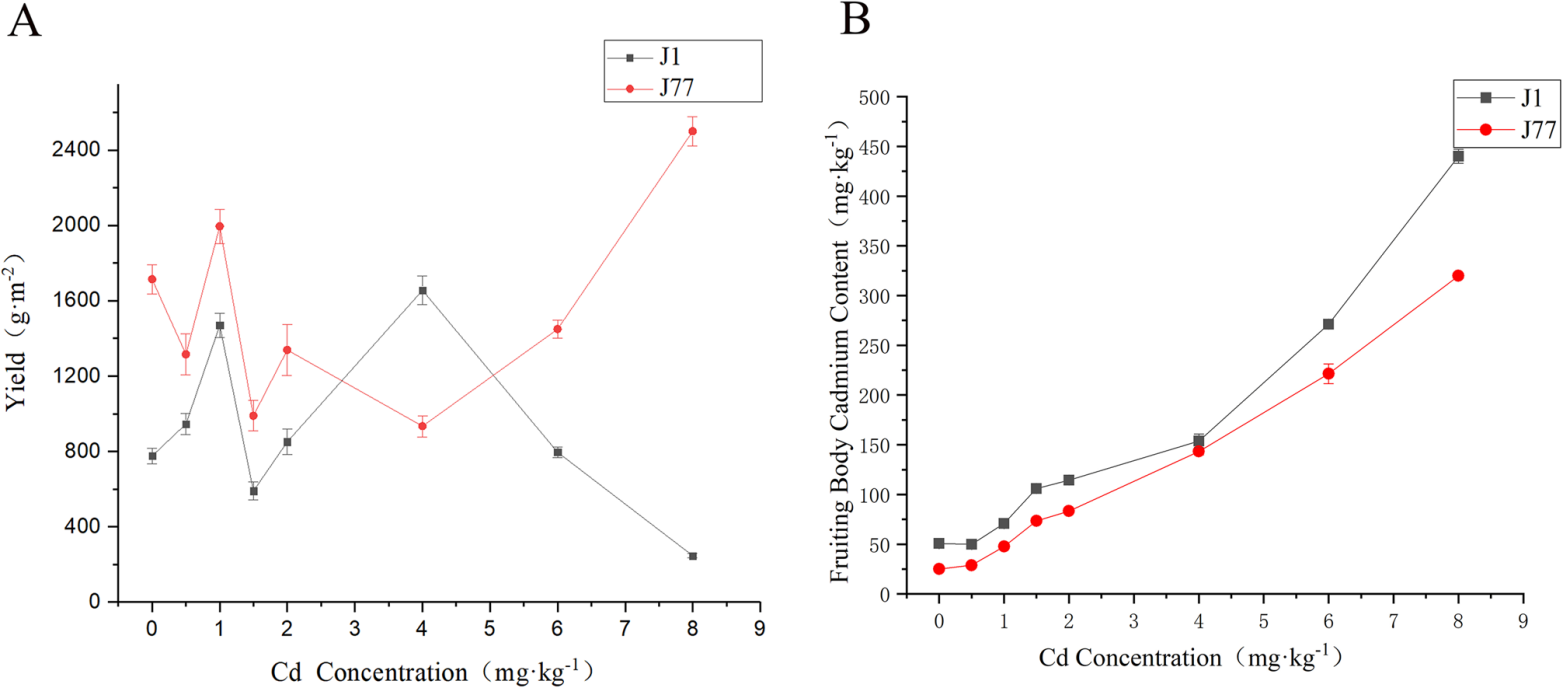
Effects of Cd stress on yield and Cd content in fruiting bodies.

It can be seen from Fig. 2A that with the increase of Cd concentration, the yield of strain J1 increased first, then decreased, then increased and then decreased. The overall trend showed that low concentration promoted the growth of strain J1. However, when the concentration increased to 6 mg kg^−1^, it showed a state of inhibition, and when the concentration increased to 8 mg kg^−1^, it showed that J1 was more sensitive to Cd. The yield of J77 increased first and then decreased. The yield of J77 was significantly higher than that of the control when the concentration increased to 8 mg kg^−1^. The results showed that J77 had strong tolerance to Cd, and high concentration of Cd could promote the growth and development of its fruiting bodies, and its yield was significantly higher than that of other treatments. The yield trends of strain J1 and J77 were consistent at low concentration. The turning point of their yield was 4 mg kg^−1^. At this turning point, J1 showed a downward trend, while J77 showed an upward trend. This indicated that there were great differences between the two strains and their tolerance to Cd stress was also different. Under high Cd stress, the yield of J1 decreased significantly, while J77 increased significantly.

### Effects of Cd stress on Cd content in fruiting bodies

It can be seen from Fig. 2B that with the increase of exogenous Cd concentration, the Cd content in both strain J1 and J77 showed an upward trend, and the Cd content in the fruiting body of J1 was always higher than J77. From the variation trend of Cd content in fruiting bodies, it can be seen that the Cd rich ability of strain J1 is much higher than J77, which verifies that strain J1 has a strong ability to enrich Cd, while strain J77 has a weak ability to enrich Cd.

### Effects of Cd stress on antioxidant enzymes in fruiting bodies

**Figure 3:**
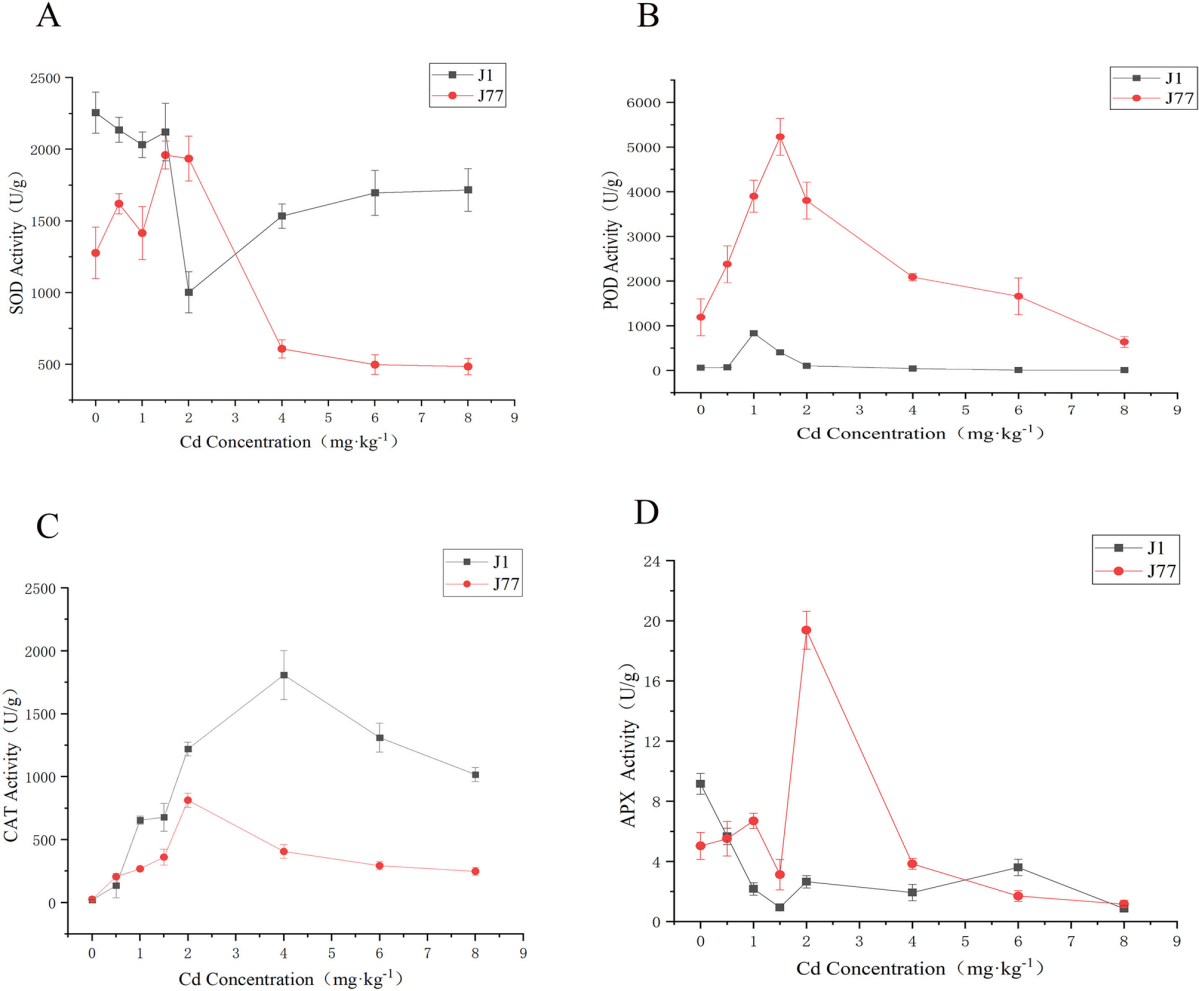
Effect of Cd stress on antioxidant enzyme activities.

It can be seen from Fig. 3A that with the increase of Cd concentration, the superoxide dismutase (SOD) of strain J1 decreased first and then increased, while SOD of strain J77 increased first and then decreased. Under 2 mg kg^−1^ Cd stress, SOD of strain J1 and J77 appeared inflection point, but the difference was that after the inflection point of J1, it showed an upward trend, while J77 showed a downward trend. This indicated that the activity of SOD of strain J1 and J77 was significantly different with the increase of Cd concentration. This difference is closely related to the characteristics of the two strains.

It can be seen from Fig. 3B that the change trend of peroxidase (POD) of J1 and J77 is relatively consistent, showing a downward trend after the peak, and the decline is more severe. The POD activity of J77 was much higher than J1, which indicated that the POD of J77 was more sensitive to Cd stress. The POD activity of J77 was the highest under 2 mg kg^−1^ Cd stress, while that of J1 was the highest at 1 mg kg^−1^ Cd stress.

With the increase of Cd concentration, the activity of catalase (CAT) first increased and then decreased. In the control group without Cd stress, the activities of both strain J1 and J77 were almost zero, indicating that the activity of CAT was relatively low under no stress (Fig. 3C). However, when the concentration increased to a certain extent, the activity of CAT decreased. The inflection point of strain J1 appeared at the concentration of 4 mg kg^−1^, which indicated that the strain had strong tolerance at this concentration. The CAT activity of strain J1 is higher than J77. The reason for this result may be that although the POD activity of J77 is very high, the proportion of CAT in J77 is too small, so the CAT activity is lower than that of strain J1.

The activity of ascorbate peroxidase (APX) is lower than other enzymes, which is mainly because it is concentrated in the chloroplast of plants, but the edible fungus do not contain chloroplast, so the activity of ascorbic acid peroxidase is lower than plants (Fig. 3D). The activity of APX of J1 changed dramatically under low concentration of Cd stress, and it changed gently at high concentration of Cd stress, while J77 was the opposite. The activity of J77 was 3.85 times higher than that of the control under 2 mg kg^−1^ Cd stress. J77 control treatment was 4.34 times higher than 8 mg kg^−1^ Cd treatment, and J1 control treatment was 10.65 times higher than J1 8 mg kg^−1^ treatment.

In conclusion, the activities of different antioxidant enzymes were different between J1 and J77, and the activities were different under different concentrations of Cd stress. The change trend of enzyme activity was mostly increased at first and then decreased, which indicated that the enzyme activity was increased under certain Cd concentration stress, and decreased when Cd stress was serious. The activities of POD and APX of J77 were higher than J1, which was one of the main reasons for the strong tolerance of J77. The activities of SOD and CAT of J1 were higher than J77, which indicated that the ability of scavenging superoxide radicals of strain J1 was stronger than J77, but the ability of resisting Cd stress was weaker than strain J77. The mechanism of different antioxidant enzymes is different, so their performance is different among different strains.

### Effects of Cd stress on malondialdehyde and proline contents in fruiting bodies

**Figure 4:**
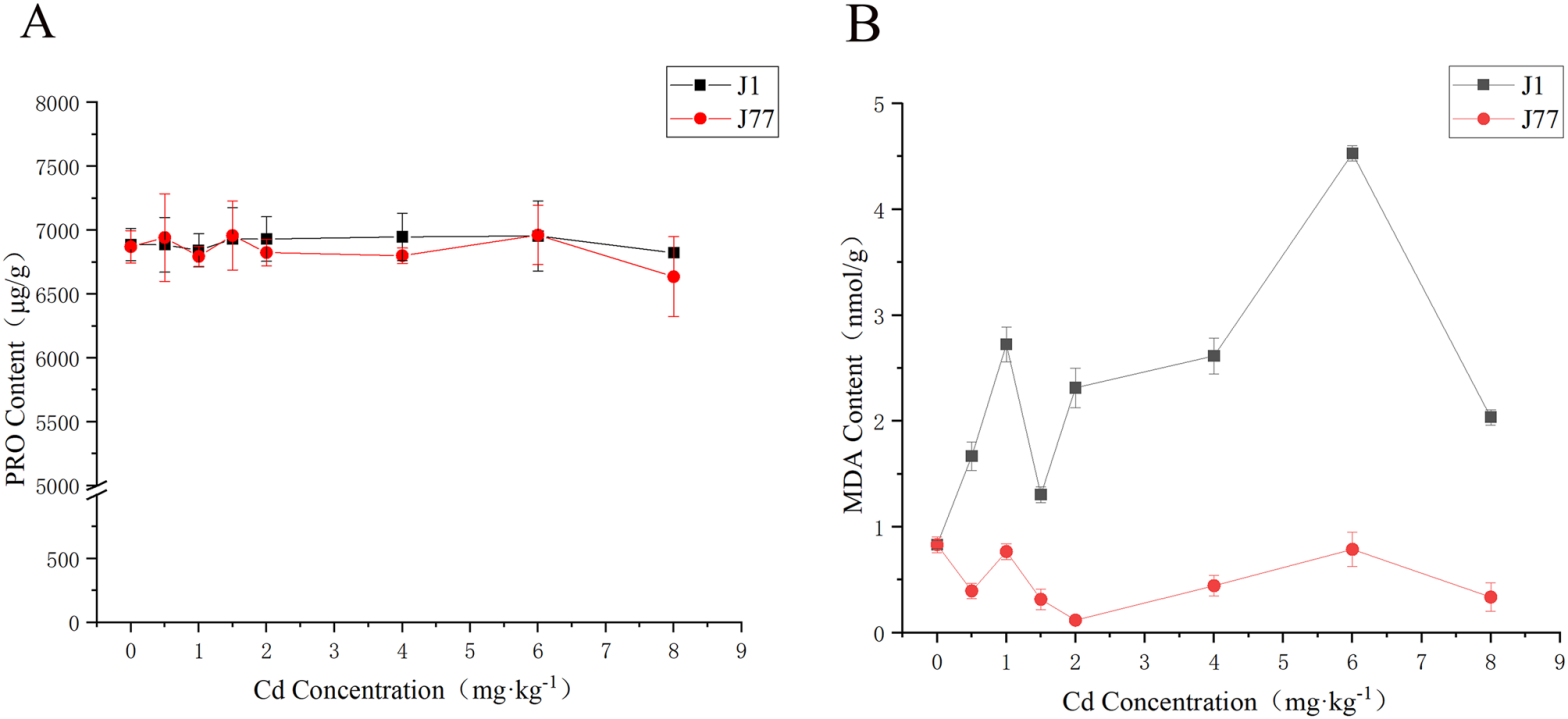
Effect of Cd stress on PRO and MDA content in fruiting bodies.

The proline (PRO) content of J1 and J77 did not change significantly, and the PRO content of J1 was higher than J77 (Fig. 4A). The two strains of J1 and J77 fluctuated in the initial stage, but not obvious in the later stage. Compared with the control, the PRO content of J77 decreased by 3.41% under 8 mg kg^−1^ Cd stress. From the figure, the PRO content of J1 and J77 is always at 7000 µg g^−1^ under different concentrations of Cd stress, which indicates that the stress resistance of J1 and J77 is strong.

The change trend of malondialdehyde (MDA) content of J1 and J77 was larger than J77, and the content of MDA in the control treatment of J1 and J77 was similar, but with the increase of Cd concentration, the content of J1 increased, while that of J77 showed a downward trend (Fig. 4B). The content of MDA accumulated in the fruiting bodies of J1 was higher than J77, and the maximum content was 5.46 multiple than J77. This fully shows that the effect of Cd stress on J1 is significantly greater than J77, which is also the reason why J1 is more sensitive to Cd stress. The MDA content of J77 was always low, so its Cd tolerance was stronger than J1.

According to the content of PRO and MDA, different concentrations of Cd stress had little effect on the content of PRO. The MDA content of J1 changed greatly, and J77 changed little. It can be seen that MDA and PRO play an important role in drought and cold resistance of plants, but have little effect on Cd stress.

### Effects of different concentrations of Cd stress on fruiting bodies quality

**Figure 5:**
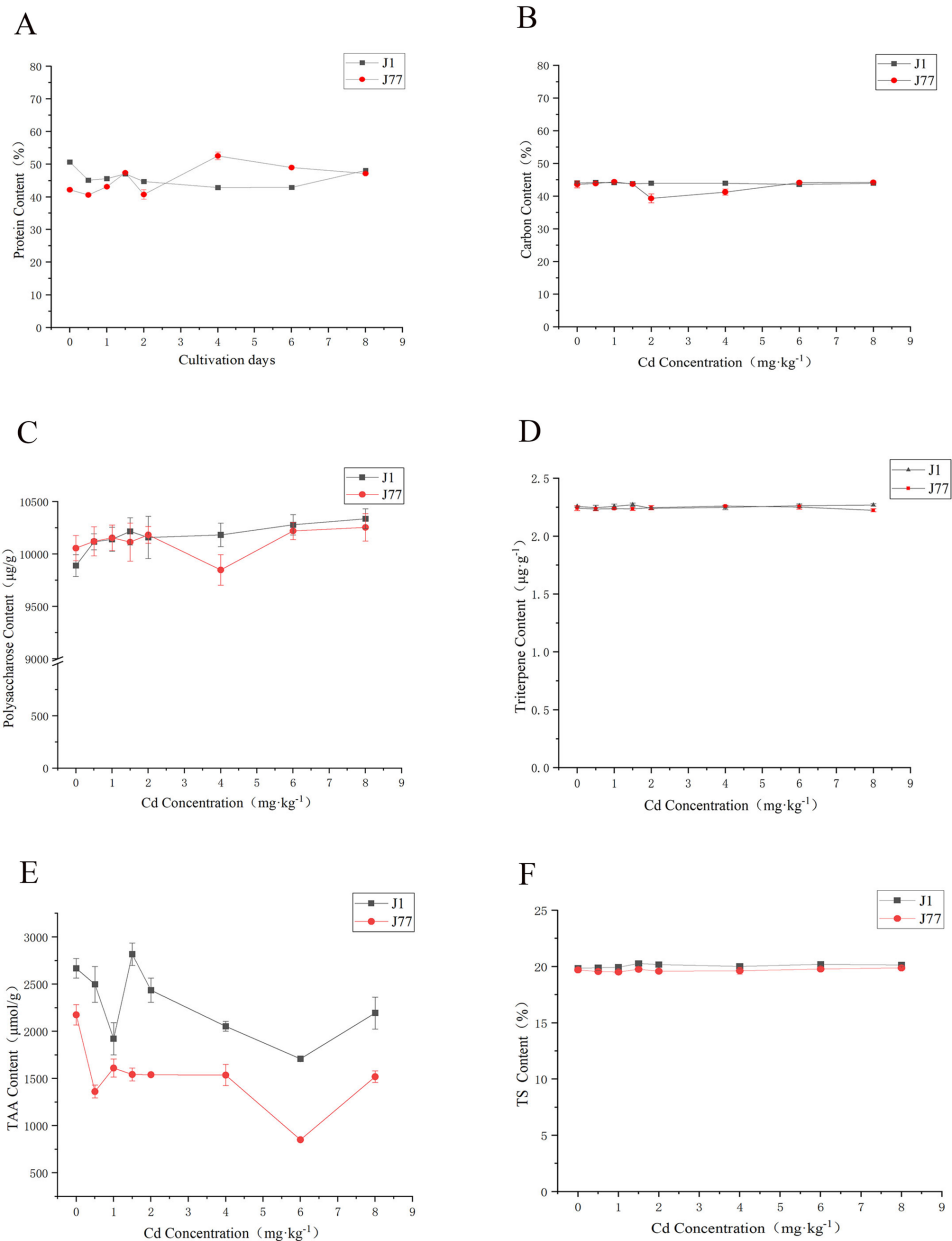
Effects of cadmium stress on nutrients in fruiting bodies A, B, C, D, E and F represent the protein, carbon, polysaccharide, triterpene, total amino acid and total sugar content in fruiting bodies of J1 and J77 under different concentrations of Cd stress, respectively.

The protein content of J1 decreased with the increase of Cd stress, while J77 increased with the increase of Cd stress (Fig. 5A). This indicated that the protein of J1 may form a chelate with Cd to resist external stress, while J77 has strong Cd tolerance, so the protein content shows an upward trend to resist the cell damage caused by Cd stress. Under the stress of 4 mg kg^−1^ Cd, the protein content of J77 was the highest, and the stress response of fruiting body was probably the strongest, and the cell damage was the most serious at this concentration.

The results showed that the carbon content of J1 fluctuated slowly, which indicated that Cd stress had little effect on carbon synthesis of J1, while J77 had no significant effect on carbon synthesis. The most obvious reduction of carbon was observed in 2 mg kg^−1^ Cd stress, which may be due to the enhanced respiration of fruiting bodies. Therefore, part of carbon was emitted into the atmosphere in the form of carbon dioxide, and the carbon content of other treatments was not significantly changed compared with the control (Fig. 5B).

With the increase of Cd concentration, J1 showed an upward trend at first, which indicated that Cd concentration might promote the accumulation of polysaccharide of J1 (Fig. 5C). Under the stress of 4 mg kg^−1^, the polysaccharide content was the lowest, which was 3.96% lower than the treatment with the highest polysaccharide content. Compared with other treatments, the control treatment of J1 showed significant difference, while the control treatment of J77 showed no significant difference with other treatments. With the increase of Cd stress, the polysaccharide content of strain J1 was different from the control treatment, which indicated that the external environment had a great influence on J1. However, J77 was stable, and the effect of external stress on its polysaccharide content was not obvious.

Different concentrations of Cd had little effect on the triterpene content of J1 and J77, and there was a certain fluctuation during the growth period, but the difference was not obvious (Fig. 5D). The content of triterpenoids in the fruiting body of *A. brasiliensis* was slightly lower, ranging from 2.22 to 2.27 μg·g^−1^. However, the content of polysaccharides was significantly higher than triterpenoids, and it was hundreds of times higher than triterpenes.

Although the variation trend of total amino acids of the two strains fluctuated greatly, the overall trend was more consistent (Fig. 5E). The total amino acids of the two strains showed a trend of first decreasing, then increasing and then decreasing. Under Cd stress, the amino acid content of strain J1 was decreased significantly. J77 strain in other treatments were significantly different from the control treatment, with a significant downward trend. The results showed that Cd stress inhibited the accumulation of total amino acids, and the differences were different under different concentrations of Cd stress.

It can be seen from Fig. 5F that there is almost no difference in the total sugar content between the two strains, but the total sugar content of strain J1 is slightly higher than that of strain J77. This is also related to the change of polysaccharide, because the total sugar mainly includes polysaccharide and monosaccharide, so when the polysaccharide content is low, the total sugar content also decreases. It can be seen from the figure that the synthesis of total sugar is not affected by Cd stress.

## Discussion

With the global heavy metal pollution becoming more and more serious, many researchers begin to focus on the harm that heavy metal pollution cause to human and plants^23^ and the use of high concentration of heavy metal plants to adsorb heavy metals in soil^24^. Among them, the problem of Cd exceeding the standard is a major problem affecting the food safety of *A. brasiliensis* industry. Heavy metal stress may be caused by oxidative stress, DNA damage, inhibition of enzyme function and destruction of protein functions that regulate cell proliferation, differentiation or apoptosis^25^. A series of changes in agronomic characters of *A. brasiliensis* were observed under Cd stress, which indicated that low concentration of Cd could promote the proliferation and differentiation of *A. brasiliensis* cells, and when the concentration was high, the cell apoptosis would be inhibited^26^. Plants absorb and transport Cd from soil through their roots^27^, however, edible fungi absorb and transport Cd from soil through hyphae, and then accumulate in stipe and pileus. In this study, there were significant differences in weight and fruiting body numbers among different treatments of J1 and J77, which indicated that Cd stress affected the physiological growth of *A. brasiliensis*. However there was no significant difference in the length of stipe and the thickness of pileus. Therefore, the two mushrooms maintained the characteristics of the original strain and did not produce much difference due to Cd stress. This indicated that although Cd stress had an impact on its growth, the strains maintained their unique characteristics.

Knowledge on the transport mechanisms of metals from the mycelium to the fruiting body is limited, and it takes place predominantly during the start of fructification. It is theorized that Cd transport is likely to be affected by sulphydryl group content in a protein carrier^28^. The results showed that the uptake of Cd by microorganisms increased with the increase of Cd concentration^29^, and application of 200 mg kg^−1^ soil Cd concentration, led to a significant accumulation of Cd in shoots and roots^30^. This is consistent with the increase of Cd concentration in *A. brasiliensis* fruiting body in this study. Different agronomic measures have certain effects on the ability of plants to absorb Cd. Taxonomically, the genus *Agaricus* seems to be highly accumulating of Cd, mainly species of the section Arvenses^31^. It is showed that metal levels reported in wild growing *A. bisporus* were considerably higher than those in cultivated fruiting bodies, probably explained not only because of the different substrate composition and pollution, but also due to the very different age of the mycelium, which may exist for several years in nature, while only for several months in a cultivation plant^32^.

Organisms use a series of different enzymes, such as SOD, POD, CAT, APX, to remove different types of ROS, thus protecting potential cell damage and tissue dysfunction. Trace metals can induce oxidative stress by inducing reactive oxygen species (ROS), and antioxidant enzymes promote reactive oxygen species resistance and clearance. SOD plays an important role in antioxidant defense by catalyzing the disproportionation of superoxide to oxygen and hydrogen peroxide^33^. SOD is an antioxidant metal enzyme existing in organism. It can catalyze the disproportionation of superoxide anion to oxygen and hydrogen peroxide. However, fungi generally contain Mn SOD and Cu/Zn SOD. Mn, Cu and Zn ions are more reductive than Cd ions, which may replace Cd to form Cd precipitation^34^. Under high concentration of Cd stress, the yield of J1 decreased, which indicated that SOD did not clear the excess free radicals in fruiting bodies in time under high concentration of Cd stress. Therefore, the activity of SOD was higher, but the yield and agronomic traits were poor. However, the high concentration of Cd in J77 increased the yield, but the activity of SOD decreased. It can be seen that SOD can remove free radicals in fruiting bodies, thus increasing the yield and decreasing the content of SOD.

When the concentration of Cd increased to a certain extent, the activity of POD showed a downward trend, which indicated that when the Cd concentration reached the limit that the fruiting bodies could bear, it would also affect the activity of POD. POD can oxidize all kinds of toxic substrates, and the result of oxidation makes these toxic substances become non-toxic substances. When J1 and J77 were exposed to Cd stress, POD could also play a role, so the activity of POD decreased under high concentration of Cd stress.

The decrease of CAT activity is one of the toxic effects of Cd. Oxygen free radicals produced under heavy metal stress inhibit these enzymes by attacking antioxidant enzymes and oxidative damage. When CAT activity decreased, the accumulation of hydrogen increased and catalase was inhibited. It is reported that the decrease of CAT activity and the increase of Cd concentration in some plants are responsible for the decrease of protein content after metal toxicity and oxidative stress^35^. In this study, the CAT activity of J1 and J77 increased with the increase of Cd concentration. When the concentration was high to a certain extent, the CAT activity showed a downward trend, but the CAT activity under stress was higher than the control. CAT activity can provide antioxidant defense mechanism for the body. Its biological function is to promote the decomposition of hydrogen peroxide in fruiting body cells, so that it will not further produce toxic hydrogen oxygen free radicals, so as to protect the function of antioxidant enzyme system, which is of great significance for the fruiting body under stress. The enhancement of CAT activity can help fruiting bodies to produce antioxidant function and resist Cd stress.

APX, a peroxidase, plays a regulatory role in intracellular ROS levels. APX is a heme peroxidase, found in all kingdoms of life, and typically catalyzes the one-and two-electron oxidation of a number of organic and inorganic substrates. Only ascorbate and cytochrome c peroxidases are typical monofunctional peroxidases with either ascorbate or cytochrome c as one-electron donor^36^. In Arabidopsis, ascorbate peroxidase 1 (APX1) also plays key roles in the reactive oxygen gene network response to abiotic stress^37^. In this study, the change of APX activity of J1 was small, and with the increase of Cd stress concentration, APX enzyme activity showed a downward trend, and the activity of each treatment was lower than the control. The APX activity of J77 increased under a certain concentration of Cd stress, and then decreased. The APX activity was inhibited by higher concentration of Cd stress.

Proline is an amino acid, which has a very beneficial effect on plants exposed to various stress conditions. The PRO concentration in cells, tissues and plant organs is regulated by the interaction of biosynthesis, degradation and intercellular transport. It is well known that PRO protects plants from stress, protects membrane integrity and stabilizes antioxidant enzymes by acting as a cell osmotic regulator between cytoplasm and vacuole, by detoxifying free radicals and buffering cell redox potential, and by stabilizing mitochondrial electron transport complex II, membranes, proteins and enzymes (such as Rubisco)^38^. The results showed that the level of ROS and other free radicals could be reduced by a certain concentration of PRO, so as to reduce the oxidative damage of plants. In addition, exogenous PRO can enhance the activity of antioxidant enzymes and affect the accumulation of endogenous plant hormones, thus improving the adaptability to various stresses. In this study, the PRO content of J1 and J77 increased with the increase of Cd concentration, which indicated that PRO content accumulated in fruiting bodies, which could better deal with the damage caused by Cd stress.

MDA is a final product of lipid peroxidation, and it has been extensively used to evaluate metal-induced oxidative stress. MDA content can be used as an indicator of the severity of stress, and the accumulation of MDA will bring certain damage to the membrane and cells, thus affecting the growth and development of fruiting bodies^39^. Oxidative stress can lead to the increase of MDA content, which is caused by membrane lipid peroxidation, which ultimately affects the function and integrity of membrane^40^. In this study, the MDA content of J1 fluctuated greatly. With the increase of Cd stress, the MDA content showed a trend of first increasing and then decreasing. After the fruiting body was stressed, the oxidative stress reaction occurred, and the MDA content accumulated. When the stress exceeded the defense ability of the fruiting bodies to a certain extent, the yield decreased significantly, and the accumulation of MDA content decreased. The MDA content of J77 fluctuated slightly, showing a trend of decreasing first and then increasing, which indicated that the effect of Cd stress on J77 strain was small, and J77 had strong resistance to stress, so the MDA content fluctuated less. The above results indicate that a decrease in invertase activity is accompanied by an increase in MDA content. The increase of reactive oxygen species will cause membrane lipid peroxidation, which will increase the content of MDA to inhibit the activity of invertase^41^.

Microorganisms can survive in high concentrations of heavy metals, which indicates that they can form an effective defense system and reduce the toxicity of heavy metals. These defense systems are based on extracellular and intracellular metabolites and can chelate with heavy metal ions. Microorganisms contain a variety of bioactive substances, such as polysaccharides, proteins and so on. These bioactive substances contain carboxyl group, phosphate group, hydroxyl group, mercaptan group, amino group and other functional groups. Through electrostatic adsorption, complexation, chelation, ion exchange and covalent adsorption, these bioactive substances combine with the surrounding heavy metal ions, or form extracellular sediments with heavy metal ions to prevent them from entering the cells^42^. Polysaccharides and proteins play an important role in antiviral activity.

Under stress conditions, plants need adaptation to survive, which ranges from simple phenotypic to complex physiochemical alteration and differential protein expression^43^. Mycorrhizal plants are able to improve their resistance against protein degradation under Cd stress and maintain natural metabolism of proteins. Stress results in a myriad of alterations in plants, like changes in soluble protein content and electrolyte leakage of the plasma membrane, which in turn decrease the efficiency of the photosynthetic apparatus giving rise eventually to a reduction of crop yield^44^. The results showed that the protein content of strain J1 decreased with the increase of Cd concentration, and increased at high concentration, which was basically the same as that of control group. The protein content of J77 increased with the increase of Cd concentration, which indicated that J77 produced a series of oxidative stress response when exposed to Cd stress, so its protein content increased to resist external stress.

Extracellular polysaccharides play a role in the response, free survival and symbiosis of legume rhizobia to zinc stress. Zinc stress can stimulate the synthesis of extracellular polysaccharide and effectively protect cells from zinc stress^45^. The polysaccharide played a vital role on enhancing tolerance of *Trichoderma asperellum* under Pb^2+^ stress. The high concentration of Pb^2+^ can promote the synthesis of polysaccharide in *Trichoderma asperellummycelia.* And the pure polysaccharide can interact with Pb^2+^ to adsorb or transform it. Under Pb^2+^ stress, the polysaccharide had response changes in chemical composition, primary structure and advance structure to reduce the amount of free Pb^2+^ and enhance the tolerance of *Trichoderma asperellum*^46^. High concentration of Cd promoted the synthesis of polysaccharide in J1. The interaction between polysaccharide and Cd resulted in the transformation of Cd, which indicated that the polysaccharide of J1 provided adsorption sites for Cd ions and reduced the toxicity of Cd to cells. The polysaccharide content of J77 increased under low concentration of Cd stress, but when the concentration increased, the polysaccharide content decreased and then increased, which indicated that the polysaccharide was more sensitive to 4 mg kg^−1^ Cd concentration.

The increase in isoleucine, phenylalanine and tyrosine under combined Cd and N stress may be because they are both glucogenic and ketogenic amino acids, while proline, histidine, glutamine, valine and asparagine on the other hand are glucogenic amino acids. The nature of the different amino acids and their possible roles in lipid and carbohydrate biosynthesis through the tricarboxylic acid cycle (TCA cycle) can explain the positive relationship they had with total lipid and carbohydrates production in *Chlorella. vulgaris*^47^. The content of total amino acids was greatly affected by Cd stress. Both J1 and J77 showed a trend of decreasing first and then increasing. This may be due to the stress response of fruiting bodies to Cd stress. The decrease of amino acid content may be due to the increase of protein synthesized in fruiting bodies and other biomolecules responding to Cd stress, so amino acids were consumed substance.

## Conclusion

There was a big difference between the two *A. brasiliensis* strains in the cultivation process. There were significant differences in physiological indexes, antioxidant enzyme activities and stress resistant substances between J1 and J77. This also confirmed that J1 was a high Cd uptake and low Cd tolerance variety, while J77 was a low Cd absorption and high Cd tolerance variety. Under high Cd stress, the yield of J1 decreased significantly, while that of J77 increased significantly. The activities of different antioxidant enzymes were different between J1 and J77, and the activities were different under different concentrations of Cd stress. The change trend of enzyme activity was mostly increased first and then decreased, which indicated that the enzyme activity was increased under certain Cd concentration stress, and decreased when the stress reached a certain degree. Different concentrations of Cd stress had little effect on PRO content. The MDA content of J1 changed greatly, and that of J77 changed little. With the increase of Cd concentration, the increase of polysaccharide of J1 was greater than that of J77. The total amino acid content of J1 was always higher than that of J77. The total sugar content of J1 was slightly higher than that of J77. There are also obvious differences between the two strains in appearance, but their quality difference is small. Therefore, it is recommended to select J77 varieties in production, which can effectively reduce the Cd content in fruiting bodies.

## Substrate and methods

### Fungal strains

*A. brasiliensis* strains J77, J1 were stored in the National Engineering Research Center of JUNCAO Technology, Fujian Agriculture and Forestry University.

### Experiments design

The Cd concentrations added to the culture materials were 0, 0.5, 1, 1.5, 2, 4, 6 and 8 mg kg^−1^ by using CdCl_2_·2.5H_2_O. The compost material was composed of 35.7% rice straw, 14.29% cow manure, 13.29% wheat bran, 35.7% rice husk, 0.02% KH_2_PO_4_ and 31% CaCO_3_. The fermentation process was as follows: the main compost was pre wetted, water was mixed and stirred, the bottom width of the pile was 1.2–1.5 m, the top width was 0.8–0.9 m, and the height was 0.8–1 m. when the compost temperature rose to 70–75 ℃ (about 7 days), the whole fermentation process was 23–25 days.

The two strains were grown on wheat grains and mixed them separately into the fermentation material, and the corresponding concentration of Cd solution was sprayed into the fermentation material^48^ After the cultivation compost are evenly mixed, transport the compost to the shelf to ensure that the thickness of the compost on the shelf is about 20 cm. Pick mature fruiting bodies, select fruiting bodies with a relatively uniform size to determine agronomic characters and store in liquid nitrogen after placing in the cryotube. Each treatment was 0.35 square meters, and each treatment was repeated three times. The mycelium can grow to 2/3 of the compost after 20 days at 24–28 ℃ during the period of germinating and covering with soil. The growth temperature of primordium and fruiting body is about 28 ℃. The fruiting body can be harvested for about 10 days from the appearance of primordium, and 4–5 stubbles can be harvested in one fruiting cycle.

### Agronomic characters and yield of fruiting bodies

Electronic scale and digital caliper were used to weigh and measure the fruiting body. The measurement indexes included: single mushroom weight and length cap diameter, stipe diameter, stipe length, cap thickness, cap weight and stipe weight.

### Cd content in fruiting bodies

Determination of Cd content in fruiting bodies by Flame Atomic Absorption Spectrometry (PerkinElmer PinAAcle 900 T)^49^.

### Antioxidant enzyme activity in fruiting bodies

Superoxide dismutase (SOD) was determined by NBT method^50^. Peroxidase (POD) was determined by guaiacol method^51^. Catalase (CAT) was determined by hydrogen peroxide method^49^. Ascorbate peroxidase (APX) was determined by spectrophotometry^36^.

### Proline and malondialdehyde content in fruiting bodies

The content of proline (PRO) was determined by acid ninhydrin method^52^. Determination of malondialdehyde (MDA) content by thiobarbituric acid method^53^.

### Fruiting bodies nutritions

Protein content was determined by Kjeldahl Nitrogen Analyzer. The content of carbon was determined by the Element Analyzer of Leco Trumac CNS (FZSYZ-8). The content of polysaccharide was determined by anthrone sulfuric acid method^54^. Triterpenoids were determined by vanillin glacial acetic acid perchloric acid colorimetry^55^. The content of total amino acids was determined by total amino acid kit^56^. The content of total sugar was determined by phenol concentrated sulfuric acid method^57^.

### Statistical analysis

Origin 2018 (StatSoft Inc., Tulsa, OK, USA) software was used to build line chart. Agronomic character determination is repeated three times for each treatment, and 10 fruiting bodies are determined for each repeat. To perform the statistical analyses, we used the SPSS statistical package (SPSS Inc., Chicago, IL, 222 USA). One-Way Analysis of Variance (ANOVA) was done. Duncan’s test was used as the post-hoc test for the separation of means (p < 0.05).
